# Optimising Antibiotic Usage to Treat Bacterial Infections

**DOI:** 10.1038/srep37853

**Published:** 2016-11-28

**Authors:** Iona K. Paterson, Andy Hoyle, Gabriela Ochoa, Craig Baker-Austin, Nick G. H. Taylor

**Affiliations:** 1 University of Stirling, Computing Science and Mathematics, Faculty of Natural Sciences, Stirling, FK9 4LA, United Kingdom; 2 Centre for Environment, Fisheries and Aquaculture Science (Cefas), Weymouth Laboratory, Weymouth, DT4 8UB, United Kingdom

## Abstract

The increase in antibiotic resistant bacteria poses a threat to the continued use of antibiotics to treat bacterial infections. The overuse and misuse of antibiotics has been identified as a significant driver in the emergence of resistance. Finding optimal treatment regimens is therefore critical in ensuring the prolonged effectiveness of these antibiotics. This study uses mathematical modelling to analyse the effect traditional treatment regimens have on the dynamics of a bacterial infection. Using a novel approach, a genetic algorithm, the study then identifies improved treatment regimens. Using a single antibiotic the genetic algorithm identifies regimens which minimise the amount of antibiotic used while maximising bacterial eradication. Although exact treatments are highly dependent on parameter values and initial bacterial load, a significant common trend is identified throughout the results. A treatment regimen consisting of a high initial dose followed by an extended tapering of doses is found to optimise the use of antibiotics. This consistently improves the success of eradicating infections, uses less antibiotic than traditional regimens and reduces the time to eradication. The use of genetic algorithms to optimise treatment regimens enables an extensive search of possible regimens, with previous regimens directing the search into regions of better performance.

The discovery of penicillin in 1928 dramatically changed human and animal health and well being. Since then, the discovery of additional antibiotics has further increased survival rates in areas such as surgery and during cancer chemotherapy. However, a lack of new antibiotics and an increase in resistance means these advances are under threat^1^. Resistance to all antibiotics in clinical use has now been observed^2^, with the extensive use and misuse of antibiotics being attributed to the spread of these resistant genes^3^,^4^,^5^. This has caused considerable debate over the future effectiveness in treating bacterial diseases^6^,^7^. As such, the World Health Organisation (WHO) has identified antibiotic resistance as one of the major health concerns of the 21st Century.

The apparent ease at which antibiotic resistance spreads is due to the ability of bacteria to acquire additional genes. Genes can be acquired via either mutations or horizontal gene transfer (HGT). While mutations are undoubtedly a source of resistance, HGT is responsible for increased propagation of resistance through bacterial populations^8^. If bacteria acquire resistant genes in an environment where they are beneficial, HGT will facilitate the spread of these genes within the population^9^. Sub-Minimum Inhibitory Concentrations (MIC) of antibiotics and the persistence of high levels of antibiotics within the environment have been linked to the emergence of resistant genes^10^,^11^. Antibiotic treatments must therefore be effective to minimise the influence of an environment which selects for resistance.

Effective antibiotic treatment regimens consist primarily of two variables: the dose and the duration of treatment. For most antibiotics, the manufacturer identifies a traditional treatment regimen which is implemented by doctors and veterinary surgeons when prescribing these antibiotics. These traditional treatment regimens usually consist of a fixed dose administered for a specified duration. Drug efficiency studies are used to determine the dose and duration for these treatment regimens. However, one limitation of this approach is that it only provides information for the regimen being analysed and offers no indication for other potential regimens. AliAbadi and Lees^12^ highlighted the importance of rational use of antibiotics and the need to incorporate population pharmacokinetic (PK) and pharmacodynamic (PD) data into dosage scheduling. While traditional treatment regimens may be effective they may not be the optimal duration or dose at which to administer antibiotics.

As the threat of antibiotic resistance spreads the need to optimise antibiotic dosage regimens becomes essential. Mathematical modelling is increasingly being used to investigate optimal treatment regimens for antibiotic therapy^13^,^14^,^15^,^16^,^17^. However, these studies either omit pharmacodynamic data, by assuming that the antibiotic induced death rate is constant; or only analyse a very limited number of alternative treatment regimens. With no verification that the duration or doses chosen are optimal, these studies look for an ‘optimal’ solution from a selection of sub-optimal treatments. This study therefore aims to address these assumptions by considering antibiotic induced death as a function of the concentration of antibiotic present and by using a genetic algorithm (GA) to identify optimal treatment regimens. The use of a GA allows for the automatic exploration of the vast space of potential treatment regimens, in order to locate the most efficient ones. The effectiveness of traditional treatment regimens in eradicating bacterial infections will be analysed and compared to the alternative treatment regimens identified using the GA. This will be the first study examining the use of a genetic algorithm to optimise antibiotic treatment regimens.

## Model

### Deterministic

In keeping with previous studies^9^,^14^,^18^,^19^ a system of coupled ordinary differential equations are used to describe the dynamics of a population of susceptible (S) and resistant (R) bacteria. As asexual reproduction requires energy it is assumed that the growth rate of bacteria is limited and therefore modelled using the standard logistic growth equation. A cost, *a*, is associated with carrying the genes which infer resistance to antibiotics^20^ and results in a reduced growth rate for the resistant strain. Genes can pass from resistant to previously susceptible bacteria through HGT, *β*, resulting in the loss of susceptible bacteria and the addition of resistant bacteria. There are 3 main mechanisms of HGT: transformation, transduction and conjugation. This study does not distinguish between the differing modes of HGT. Both susceptible and resistant bacteria die at a natural death rate, *θ*, and through exposure to antibiotics, *A*_*i*_(*C*).

Antibiotics are added to the system in daily doses. When 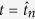 the concentration of antibiotic *C*(*t*) = *C*(*t*) + *D*_*n*_, where 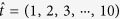 and *D* is a vector of doses *D* = (*D*_1_, *D*_2_, …, *D*_10_). Traditional treatment regimens assume that *D*_1_ = *D*_2_ = … = *D*_10_, however this study relaxes this constraint. Treatment regimens within this study are limited to a maximum of 10 doses but this could be increased indefinitely. The full model can therefore be written as equations 1, 2 and 3. A full list of parameters and values can be found in Table 5 (see methods section).


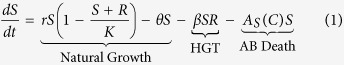



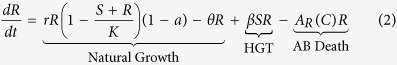



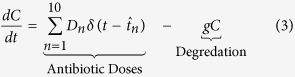


### Stochastic

Deterministic modelling contains no randomness and as a result produces the same outcome each time it is run. Provided the population densities are not too small, deterministic models produce good approximations of the system dynamics. However hosts treated with the same treatment regimen will not all respond in exactly the same way. The small population size of resistant bacteria mean that stochastic events may lead to the emergence or extinction of a resistant strain. A stochastic framework was therefore produced for equations 1, 2 and 3. Simulations are run for 30 days to allow for infection to return if treatment regimens are unsuccessful. Each treatment regimen was run 5000 times with the infection either being eradicated or still present at the end of the 30 days. The percentage of runs which resulted in eradication of the infection produced the success rate for each regimen. Due to the ability to simulate the model thousands of times the variability within the results is small. However, the 95% confidence interval for each treatment was calculated in MATLAB using the Clopper-Pearson exact confidence interval^21^. This method was chosen due the occurrence of success rates close to 100%. The median time to eradication for all successfully eradicated infections was also calculated. (This data was not normally distributed and therefore the median was used instead of the mean).

## Results

Numerical simulations were run to analyse the effect different treatment regimens have on the population size of bacteria within an infection. The success rate and time to eradication of the infection were analysed. Treatment regimens are obtained from traditional regimens and from solutions derived using a GA. The results presented were performed with an initial resistant population of 10% of the total bacterial population. When analysed with an initial resistant population of 1% of the total bacterial population the results follow a similar pattern (see Supplementary Table S1).

### Traditional Treatment Regimens

**Figure 1:**
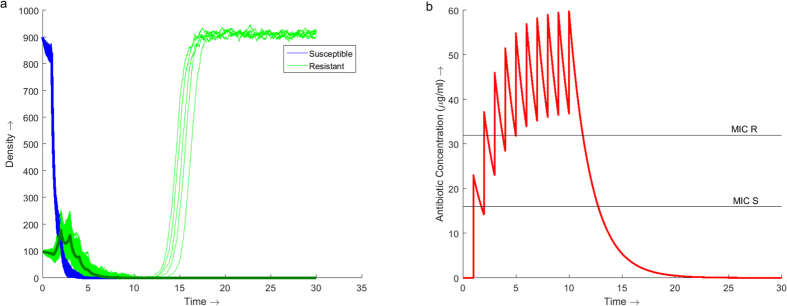
Dynamics of the model over 30 days with antibiotic therapy administered at a daily dose of 23 *μg*/*ml* for the first 10 days. (**a**) Stochastic simulations of the population dynamics of both susceptible (blue) and resistant (green) bacteria with the deterministic dynamics (bold) overlaid. 5000 simulations were run producing a success rate of eradicating the infection of 99.8% (95% CI: 99.6, 99.9). (**b**) Simulation of the concentration profile of antibiotic present within the system over the 30 day duration. The MIC lines indicate the concentration of antibiotic required to inhibit the growth of the respective bacterial strain, 16 *μg*/*ml* for susceptible bacteria and 32 *μg*/*ml* for resistant bacteria. A maximum antibiotic concentration of 60 *μg*/*ml* is observed on Day 10.

Using traditional treatment strategies of a constant dose administered for 10 days the minimum daily dose required to successfully treat the infection is 23 *μg*/*ml* (Fig. 1). Under this regimen the infection is successfully eradicated in 99.8% (95% CI: 99.6, 99.9) of cases (n = 5000 for all simulations). Administering 23 *μg*/*ml* of antibiotics per day increases the concentration of antibiotic within the system over the 10 days, reaching a peak of 60 *μg*/*ml* on day 10 (Fig. 1b).

From Fig. 1b it is noted that it takes 3 days before the concentration of antibiotic is maintained above the MIC of the resistant strain. During these first 3 days the population of resistant bacteria increases (Fig. 1a). Once above the MIC of the resistant strain the population begins to decrease. If the infection is not eradicated under the traditional treatment regimen then a resistant infection will emerge.

Until now the study assumed that traditional treatment regimens are administered over 10 days. This assumption was relaxed and the success rate of eradicating the infection over a shorter duration examined (Table 1). Shorter treatment duration results in a decrease in the success rate of eradicating the infection. Treatment duration fewer than 8 days experiences a substantial decrease in success rate, to below 90%.

The time taken to eradicate the bacterial population was also measured. This time was only recorded in the cases where the treatment was successful and the bacterial population completely eradicated. There is a small decrease in the time to eradication as the treatment duration decreases from 10 days to 7 days. However, this is due to the shorter regimen leading to a lower success rate. The 7 day traditional treatment is unable to eradicate infections which persist beyond 8 days due to the antibiotic continuously degrading beyond the last day of treatment. Due to these persistent infections not being eradicated the median time to eradication lowers in comparison to the longer traditional treatment regimens. As the treatment length increases above 7 days the success rate also increases. The median increase in success rate from 8 days to 10 days is 3.4% but requires 18.7% more antibiotic to achieve this. To maintain a success rate of over 90%, under a traditional treatment regimen, this infection can be treated by administering a minimum of 184 *μg*/*ml* of antibiotic over 8 days. This regimen results in a success rate of 96.4% and is used as the baseline to look for improved treatments.

### Tailored Treatment Regimens

A genetic algorithm (GA) was used to identify effective dosage vectors, *D* = (*D*_1_, *D*_2_, …, *D*_10_), which would maximise the success rate of eradicating the infection by minimising the fitness (objective) function (Eq. 4).


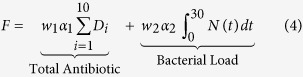


Minimising the total amount of antibiotic used, ∑_*i*_*D*_*i*_, exposes the environment to less antibiotic reducing the likelihood of resistance developing. However, using less antibiotic increases the total bacterial burden on the host over the length of the infection, 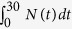, where *N* = *S* + *R*. The increased bacterial load not only compromises the health of the host but also offers more opportunity for mutations to arise increasing the risk of further resistance developing. A trade-off exists between the total amount of antibiotic used and the total bacterial load over the course of the infection. Weights *w*_1_ and *w*_2_ allow for more emphasis to be placed on minimising one term over the other. To ensure a trade-off exists, 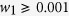 and 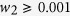 (However, this study later considers the case where *w*_1_ = 0, hence the objective is solely to maximise treatment success.) Due to the difference in the magnitude of the values of each term, correcting factors *α*_1_ and *α*_2_ were used to transform the terms between 0 and 1.

### Genetic Algorithm with the Deterministic Model

Due to the toxic nature of antibiotics the total antibiotic concentration within the system at any point in time was constrained to a maximum of 60 *μg*/*ml* within the GA. This is in keeping with the maximum concentration from the traditional treatment regimen (although this could be relaxed if needed). The GA was run for varying maximum daily dosages of 60, 50 and 40 *μg*/*ml* per day. The successful dosage vectors were then run through a stochastic model to generate a success rate of eradicating the infection.

The dosage vectors from the GA begin with an increased dose which tapers off as the treatment progresses (Table 2). Results from the GA suggests that the duration of therapy could be as little as 4 days (Table 2, regimens *D1* and *D3*). However, these treatment regimens have a lower success rate, 91.2% (95% CI: 91.0, 92.5) and 92.3% (95% CI: 91.5, 93.0), than the traditional regimen, 96.4% (95% CI: 95.8, 96.9). For all three maximum daily doses, the longer duration regimens (Table 2, regimens *D2*, *D5* and *D8*) are more efficient at treating the infection than the shorter durations with success rates of 94.3% (95% CI: 93.6, 94.9), 94.4% (95% CI: 93.7, 95.0) and 95% (95% CI: 94.4, 95.6) respectively. The lack of noise within the deterministic model allows the GA to be very effective in minimising the total antibiotic used. When the shorter dosage vectors from the GA using the deterministic model are analysed using the stochastic model there is too little antibiotic administered over too short a duration leading to the emergence of resistant bacteria.

**Figure 2:**
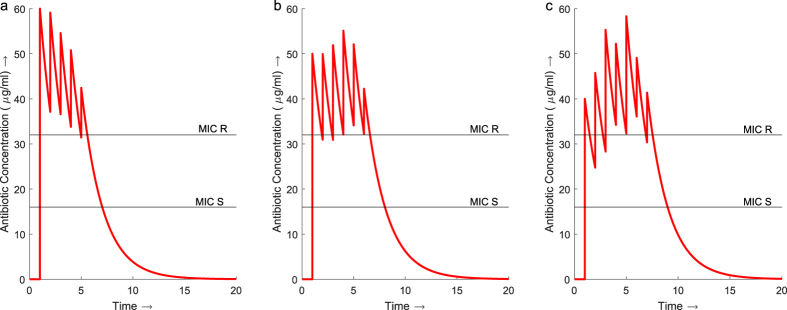
Concentration profiles for regimens *D2*, *D5* and *D8* from the dosage vectors identified by the GA with deterministic modelling. (**a**) Treatment regimen *D2* maintains an antibiotic concentration above the MIC of the resistant strain throughout the 6 day treatment. The maximum total concentration of antibiotic is 60 *μg*/*ml*. (**b**) *D5* also maintains a concentration above the MIC for the resistant bacteria throughout the 6 day treatment reaching a maximum total concentration of 54 *μg*/*ml* on day 4. (**c**) The concentration of antibiotic throughout *D8* increases above the MIC of the resistant bacteria initially but drops back below for the first two days. The concentration is then maintained above the resistant MIC for the remainder of the treatment, reaching a maximum concentration of 58 *μg*/*ml* on day 5.

The total concentration of antibiotic in the traditional regimen (Fig. 1b) increases slowly over the 8 days. The regimens from the GA start with an initial high dose followed by tapering smaller doses which maintain the total concentration of antibiotic above the MIC of the resistant bacteria for the majority of the duration of treatment (Fig. 2). All three regimens *D2*, *D5* and *D8* use less antibiotic in total over a shorter duration than the traditional regimen. Regimen *D2* uses 30% less antibiotic over 5 days instead of 8. Regimen *D5* produces a dosage vector which uses 23% less antibiotic than the traditional regimen and delivers it over 6 days instead of 8. The dosage vector from *D8* uses 15% less antibiotic and is shorter by 1 day in duration.

All the regimens identified by the GA see a reduction in the time to eradication for the infection. The median time to eradication for the 8 day traditional treatment was 7.13 days (95% CI: 7.04, 7.20). By distributing the antibiotic in a high initial dose with tapering smaller doses the median time to eradication for all the the regimens identified by the GA is between 4 and 5.5 days.

### Genetic Algorithm with the Stochastic Model

The GA was run using a stochastic model to maximise the probability of eradication and explore the effectiveness of a longer treatment duration. For the GA using the stochastic model the second term, minimising the bacterial load, in F (Eq. 4) was replaced with a term minimising the number of unsuccessful runs out of the 5000. Due to the increased run time, only a few results could be given (Table 3).

The dosage vectors from the stochastic model are noisy due to the randomness in the model. Despite this, the dosage vectors begin to converge to a similar pattern identified using the GA with the deterministic model. A large initial dose followed by an extended period of tapering lower doses is observed. The median time to eradication for the stochastic results are comparable to the deterministic results. However, by using more antibiotic over the longer treatment duration the stochastic regimens have a greater success rate. Despite the increase in total antibiotic these dosage vectors use between 11 and 19% less antibiotic than the traditional regimen with a similar or increased success rate. Dosage regimen *S2* has the greatest success rate, 98.4% (95% CI: 97.7, 98.5), an increase on the traditional 8 day treatment, 96.4% (95% CI: 95.8, 96.9). The GA was able to identify alternative treatment regimens using less antibiotic with a success rate of eradication equal to or better than the traditional treatment. The alternative regimens also successfully treat the infection over a shorter duration than the traditional regimen, around 4 to 5 days, vs. 7 to 7.5 days respectively.

If the priority is not to reduce the total antibiotic used, the GA can be implemented to maximise the effectiveness of current regimens. In this case, how can the 184 *μg*/*ml* of antibiotics be distributed to maximise the probability of eradication? (i.e. set *w*_1_ = 0 in Eq. 4) The GA identifies a high initial dose followed by a tapering of doses (Table 3, regimen *S4*) as the optimal distribution of the antibiotics. This regimen resulted in a success rate of 99.7% (95% CI: 99.5, 99.8) compared to 96.4% (95% CI: 95.8, 96.9) obtained from the traditional treatment (Table 1). This regimen also eradicates the infection quicker than the traditional regimen with a median time to eradication of 3.94 days (95% CI: 3.89, 3.99) compared to 7.13 days (95% CI: 7.04, 7.19) for the traditional regimen.

### Sensitivity Analysis

**Figure 3:**
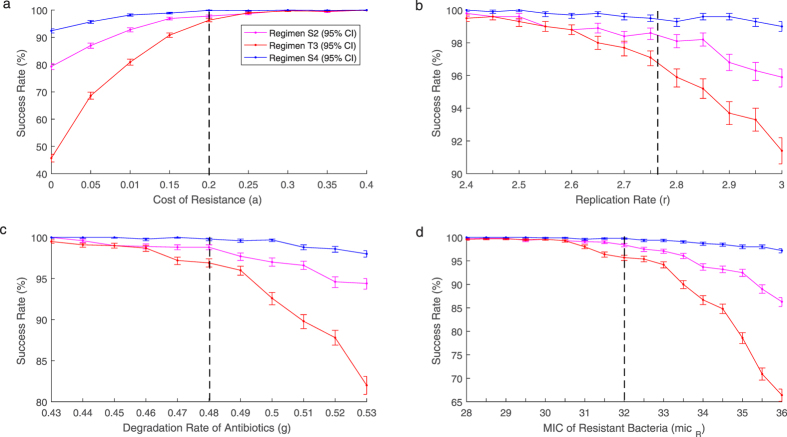
Success rates for regimens *S2* (pink), *T3* (red) and *S4* (blue) at varying values for parameters (**a**) *a*, (**b**) *r*, (**c**) *g* and (**d**) *mic*_*R*_. Black dashed line shows original parameter values. As parameter values are altered to benefit the infection success rates for all three treatment regimens decrease. With the tapered regimens performing better than the traditional regimen. If parameter values are altered to disadvantage the infection the three regimens converge to a similar success rate.

Due to the difficulty in obtaining exact parameter values for an infection, the effect changes in parameter values have on the success rate of different treatment regimens was analysed. Parameter values relating to the virulence of the bacteria; replication rate (*r*), transmission rate (*β*) and cost of resistance (*a*) were examined. Further sensitivity analysis was performed for parameters concerning the effectiveness of the antibioitcs: degradation rate (*g*), MIC of susceptible (*mic*_*S*_) and resistant bacteria (*mic*_*R*_) and the shape of the antibiotic death function (*k*). Changes in parameters *r*, *a*, *g* and *mic*_*R*_ show the greatest change and can be found in Figure 3. Other results can be found in Supplementary Figure S1. Analysis was performed on the traditional 8 day treatment regimen (Table 1, regimen *T3*) and GA generated treatment regimens (Table 3, regimens *S2* and *S4*).

As *r*, *g* and *mic*_*R*_ decrease, the success rate for all three treatment regimens converge towards 100%. At these lower parameter values the tapered regimens have no benefit over the traditional regimen. However, as *r*, *g* and *mic*_*R*_ increase the success rates for all 3 treatments decrease. As the parameter values continue to increase the benefit of the new tapered regimens increase significantly over the traditional regimen. The cost of resistance follows a similar pattern. As *a* increases the three treatment regimens are equally as effective with all success rates converging to 100%. However, when *a* is decreased the success rates for all three treatments also decrease. Despite the decrease in success rates the tapered regimens obtained from the GA perform better than the traditional regimen. When there is no cost of resistance the success rate of the traditional regimen dropped to below 50% at 45.7% (95% CI: 44.3, 47.1) whereas the tapered regimens remain significantly higher at 79.3% (95% CI: 78.2, 80.4) and 92.4%(95% CI: 91.6, 93.1). Across all the parameter values analysed regimen *S4* consistently maintains a success rate above 90%. Whereas when the same amount of antibiotic is distributed in a traditional manner the success rate can drop to below 50%. Despite regimen *S2* using less antibiotic it also consistently performs better than the traditional regimen.

While the previous tapered regimens perform well when the parameter values are altered, they are not necessarily the optimal dosage vectors for these new parameter sets. To examine whether the tapered effect was a consequence of the parameter values chosen the GA was used to generate optimal dosage vectors for the varied parameter values found in Fig. 3. In every run of the GA, the optimal solution was an initial high dose followed by tapering doses. Although the optimal solutions do not change qualitatively, i.e. high dose with tapering, the exact doses do vary substantially. An example is shown in Table 4 where the growth rate was varied by 10%. Here the same pattern holds qualitatively but there was variation in the exact doses. Tapered regimens may be optimal, however the exact doses need to be personalised across infections.

## Discussion

Current antibiotic treatment regimens consist of a fixed daily dose administered for a set duration. While these traditional regimens may be easier to administer due to the constant dose, there is little evidence that this is the optimal way of administering antibiotics. Despite the continued increase in antibiotic resistance these traditional treatment regimens remain largely unchanged. More research must be dedicated to ensuring we are using antibiotics in an optimal way.

This study considered a traditional treatment regimen of 23 *μg*/*ml* of antibiotic per day for 10 days. While this regimen successfully eradicated the infection in 99.8% of cases, the daily dose of antibiotic falls between the MIC of the susceptible and resistant bacteria initially facilitating the emergence of resistance. This is due to the time it takes for the total concentration of antibiotic to increase above the MIC of the resistant strain. While between the two MIC points the susceptible bacteria are eradicated allowing the resistant population to increase with little competition. Provided treatment is continued, the concentration of antibiotic will eventually increase above the MIC of the resistant strain.

The GA, using the deterministic and stochastic models, identified that optimal dosage vectors contain an initial high dose followed by tapering lower doses. Initially increasing the concentration of antibiotic above the MIC of the resistant bacteria eradicates the selective advantage observed in the traditional treatment regimen. Smaller doses of antibiotic are then administered to maintain the concentration above the MIC of the resistant strain. In the example shown the tapered regimens reduce the amount of antibiotic required to successfully treat the infection by as much as 23%. In some regimens produced by the GA, the maximum concentration of antibiotic within the system was lower than that observed with the traditional treatment regimen (Fig. 2) despite prescribing higher doses. With increased levels of antibiotic selecting for increased resistance, the ability to successfully treat an infection while maintaining a lower total antibiotic concentration over a shorter duration minimises the risk of higher resistance being selected for.

If traditional treatment regimens are adapted to deliver doses above the MIC of the resistant strain then the initial facilitation of resistant bacteria will disappear. However, the total antibiotic concentration within the system will considerably increase. In this scenario increasing the daily dose to above that of the MIC of the resistant strain would increase the total antibiotic concentration to beyond the level determined as toxic, 60 *μg*/*ml*, after 3 days.

The set duration of treatment is often subjective with increased length being used as a precaution. Studies have looked to find the optimal length of therapy^22^,^23^,^24^ but potential treatment durations are based on empirical evidence. This study used a mathematical model as a way to determine the time to eradication of the infection and therefore the minimum duration of treatment required. The 10 day traditional treatment has a median time to eradication of 7.31 days. Additional antibiotic treatment beyond 8 days resulted in a small increase in success rate despite a larger increase in total antibiotic required. Whereas treatment length fewer than 8 days (shorter than the time to eradication) resulted in a much lower success rate. A traditional treatment regimen of 23 *μg*/*ml* of antibiotic per day for 8 days was therefore taken as a baseline treatment.

The GA can be used to redistribute the antibiotic within the traditional regimen to produce a more efficient treatment regimen. The 8 day traditional treatment used 184 *μg*/*ml* of antibiotics and achieved a success rate of 96.9% with a time to eradication of 7.13 days. The alternative treatment regimen identified by the GA applied the 184 *μg*/*ml* of antibiotic in a high dose tapered regimen to achieve a success rate of 99.7% with a time to eradication of 3.94 days. This success rate is comparable with the success rate for the 10 day traditional treatment but the GA generated regimen uses 20% less antibiotic over fewer days to achieve it. By redistributing the antibiotic in a high dose, tapered pattern the time to eradication of the infection reduces considerably, allowing shorter treatment regimens to be just as effective.

Studies have shown that shorter treatment regimens can be effective in treating bacterial infections^25^,^26^ with initial loading dose treatments being beneficial in treating patients in critical care medicine^27^. Tapered regimens have been found to be effective when treating *Clostridium difficile*^28^,^29^. However, the use of tapered regimens resulted in sub-optimal performance in previous studies using optimal control strategies^30^,^31^. From the sensitivity analysis it is shown that as the parameters are altered it is possible for the success rate of a tapered regimen to drop significantly. In the case of reducing the cost of resistance the success rate for the tapered regimen dropped to below 80%. However, when the GA is used to identify an optimal solution for the new parameter set it produces the same tapered pattern but with different dose values. Generic tapering regimens will not always be the most efficient regimen. The sensitivity in the doses required for a successful tapering regimen indicates that personalisation of individual treatments is required. Such personalisation can be achieved with the use of a GA. Despite the need for personalisation the tapered regimens consistently performed better than the traditional regimen when the infection was more virulent or the antibiotic was less effective.

Using a GA to search for an effective treatment regimen allowed for a constrained search of all possible dosage vectors. The lack of noise within the deterministic model allows the GA to converge to a specific minimum antibiotic concentration. However, when this is analysed using the stochastic model, random events mean these treatments are not as efficient. The stochastic model therefore identifies slightly longer treatments with more antibiotic than the deterministic model, increasing the success rate. The increased computation time of the GA using the stochastic model makes it inefficient. The GA using the deterministic model is much less computationally expensive and produces the same loading dose but with a shorter tapered duration than the results using the stochastic model. The results from the deterministic model still have value and provide a suitable starting point from which to base potential future treatment regimens.

The use of the GA suggests that, in order to optimise antibiotic treatment regimens, the idea of constant doses needs to be addressed. Research indicates that the use of combination or sequential treatments are more effective in preventing resistance^13^,^15^,^32^. However, these studies use sub-optimal traditional treatments as comparisons and therefore single antibiotic treatments should not be ruled out. Genetic algorithms provide an efficient way of identifying and investigating the potential use of alternative single, and multiple, antibiotic treatment regimens to prolong the effectiveness of current antibiotics.

## Methods

### Antibiotic Death Rate

Experimental data^33^,^34^ suggests that as the concentration of antibiotic increases the death rate increases until it reaches a saturation point. In addition, the concentration of antibiotic naturally decays within a host. The concentration of antibiotic is therefore modelled according to first order kinetics with an elimination constant *g*. The half-life of the antibiotic is therefore given by 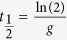.

To model the relationship between antibiotic concentration and antibiotic induced death rate the extension of the Emax model of antibiotic treatment by Regoes *et al*.^30^ was used (Eq. 5).


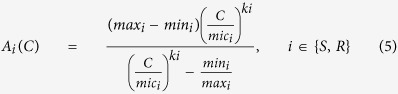


### Parameter Values

The parameter values (Table 5) were chosen such that in the absence of antibiotics the resistant strain would not out-compete the susceptible strain. Analytical analysis of the model was performed to identify the conditions which meet this criteria (see Supplementary Equations). This ensures that if resistance invades it is due to the treatment regimen and not a result of the dynamics of the system.

### Stochastic Modelling

This study uses the well-established Gillespie algorithm^35^ to obtain stochastic simulations for the different treatment regimens. By calculating the probability of the individual events occurring, based on rates and parameter values from the deterministic model, the Gillespie algorithm randomly chooses the next event to happen and the time at which it will happen. The population of each bacteria is adjusted accordingly and the process is repeated. As the events are chosen randomly each simulation will be slightly different. The success rate for each treatment regimen was obtained by calculating the total number of simulations which resulted in the eradication of both susceptible and resistant bacteria.

### Genetic Algorithms

Genetic algorithms were proposed by John Holland in the early 1970’s^36^. They belong to the larger class of evolutionary algorithms, which generate solutions to optimisation problems using techniques inspired by natural evolution, such as inheritance, mutation, selection and crossover^37^. GAs have previously been used to generate treatment schedules for chemotherapy treatment^38^,^39^. Despite being a randomised search GAs are by no means random, instead they use historical information to direct the search into the region of better performance within the search space. The GA uses the deterministic model to run simulations using the generated dosage vectors. Values from these simulations are then used to compute the fitness function for that dosage vector. The fitness function of each generated dosage vector are compared with the search space moving towards the vector with the smallest fitness function. (The GA was implemented using MATLAB with a population size of 100, for 1000 generations and repeated 50 times with values of 0.01 and 0.99 for Eq. 4, *w*_1_ and *w*_2_, respectively). Varying the weights had no significant effect on the results (see Supplementary Table S2). Solutions were then run through the Gillespie algorithm to produce a success rate of eradication for each vector.

An alternative approach is to use the stochastic model as part of the fitness function evaluation within the GA. However, the computational time increases substantially (in the order of 10^3^) compared to using the deterministic model.

## Additional Information

**How to cite this article**: Paterson, I. K. *et al*. Optimising Antibiotic Usage to Treat Bacterial Infections. *Sci. Rep.* **6**, 37853; doi: 10.1038/srep37853 (2016).

**Publisher's note:** Springer Nature remains neutral with regard to jurisdictional claims in published maps and institutional affiliations.

## Supplementary Material

Supplementary Information

## Figures and Tables

**Table 1:** Comparison of success rate and time to eradication for traditional treatment dosage vectors of varying duration.

	Dosage Vector	Total Antibiotic	Success Rate (%) [95% CI, n = 5000]	Time to Eradication (days) [95% CI]
*T1*	(23, 23, 23, 23, 23,23, 23, 23, 23, 23)	230 *μg*/*ml*	99.8 [99.6, 99.9]	7.31 [7.23, 7.39]
*T2*	(23, 23, 23, 23, 23,23, 23, 23, 23, 0)	207 *μg*/*ml*	99.0 [98.7, 99.3]	7.29 [7.19, 7.35]
*T3*	(23, 23, 23, 23, 23,23, 23, 23, 0, 0)	184 *μg*/*ml*	96.4 [95.8, 96.9]	7.13 [7.04, 7.19]
*T4*	(23, 23, 23, 23, 23,23, 23, 0, 0, 0)	161 *μg*/*ml*	87.4 [86.4, 88.3]	7.12 [7.04, 7.20]

For time to eradication of regimens *T1*, *T2*, *T3* and *T4*; n = 4990, 4950, 4820 and 4370 respectively.

**Table 2:** Comparison of dosage vectors produced by the GA with deterministic modelling.

	Dosage Vector	Total Antibiotic	Success Rate (%) [95% CI, n = 5000]	Time to Eradication (days) [95% CI]
*D1*	(60, 21, 22, 15, 0,0, 0, 0, 0, 0)	118 *μg*/*ml*	91.2 [91.0, 92.5]	3.93 [3.88, 3.99]
*D2*	(60, 22, 18, 17, 11,0, 0, 0, 0, 0)	128 *μg*/*ml*	94.3 [93.6, 94.9]	3.98 [3.94, 4.04]
*D3*	(50, 29, 22, 21, 0,0, 0, 0, 0, 0)	122 *μg*/*ml*	92.3 [91.5, 93.0]	4.12 [4.06, 4.17]
*D4*	(50, 28, 20, 20, 10,0, 0, 0, 0, 0)	128 *μg*/*ml*	93.2 [92.5, 93.9]	4.17 [4.11, 4.23]
*D5*	(50, 19, 21, 23, 18,10, 0, 0, 0, 0)	141 *μg*/*ml*	94.4 [93.7, 95.0]	4.56 [4.50, 4.64]
*D6*	(40, 35, 23, 21, 13,0, 0, 0, 0, 0)	132 *μg*/*ml*	92.5 [91.7, 93.2]	4.46 [4.41, 4.51]
*D7*	(40, 26, 26, 23, 17,11, 0, 0, 0, 0)	143 *μg*/*ml*	94.0 [93.2, 94.5]	4.77 [4.71, 4.86]
*D8*	(40, 21, 27, 18, 26,13, 11, 0, 0, 0)	156 *μg*/*ml*	95.0 [94.4, 95.6]	5.33 [5.26, 5.41]

Regimens *D1*, *D3* and *D6* represent the best dosage vectors with maximum daily doses of 60, 50 and 40 *μg*/*ml* respectively. All other runs represent the best dosage vector of increased treatment duration. For Regimens *D1*–*D8*; n = 4560, 4715, 4615, 4660, 4720, 4625, 4700 and 4750 respectively.

**Table 3:** Comparison of dosage vectors produced by the GA with stochastic modelling for maximum daily doses of 60, 50 and 40 *μg*/*ml* and the case where all 184 *μg*/*ml* of antibiotic is used.

	Dosage Vector	Total Antibiotic	Success Rate (%) [95% CI, n = 5000]	Time to Eradication (days) [95% CI]
*S1*	(60, 19, 17, 16, 19,18, 0, 0, 0, 0)	149 *μg*/*ml*	96.9 [96.2, 97.2]	4.14 [4.09, 4.20]
*S2*	(50, 25, 24, 20, 20,12, 0, 0, 0, 0)	151 *μg*/*ml*	98.4 [97.7, 98.5]	4.23 [4.18, 4.31]
*S3*	(40, 27, 21, 22, 23,12, 18, 0, 0, 0)	163 *μg*/*ml*	97.1 [96.6, 97.5]	5.03 [4.96, 5.11]
*S4*	(60, 22, 22, 22, 18,15, 14, 11, 0, 0)	184 *μg*/*ml*	99.7 [99.5, 99.8]	3.94 [3.89, 3.99]

n = 4845, 4920, 4855 and 4985 for time to eradication of *S1*, *S2*, *S3* and *S4* respectively.

**Table 4:** Optimal dosage vectors achieved when growth rate is altered by ±10%.

Parameter	Value	Dosage Vector	Total Antibiotic
*r*	2.5	(60, 21, 16, 16, 17, 13, 0, 0, 0, 0)	143 *μg*/*ml*
	2.7	(50, 19, 21, 23, 18, 10, 0, 0, 0, 0)	141 *μg*/*ml*
	3	(44, 32, 23, 14, 18, 0, 0, 0, 0, 0)	131 *μg*/*ml*

**Table 5:** List of parameters and values.

Parameter	Description	Value
*r*	Replication Rate	2.7726
*K*	Carrying Capacity	1000
*β*	Rate of Transmission of Resistant Plasmid	0.00001
*θ*	Natural Death Rate	0.2
*a*	Cost of Resistance	0.2
*g*	Degradation rate of antibiotic	0.48
*min*_*S*_	Min net growth at high AB concentrations	−2.1
*max*_*S*_	Max net growth in absence of AB	*r* − *θ*
*mic*_*S*_	Min inhibitory concentration (MIC)	16
*k*_*S*_	Hill coefficient	4
*min*_*R*_	Min net growth at high AB concentrations	−2.1
*max*_*R*_	Max net growth in absence of AB	*r*(1 − *a*) − *θ*
*mic*_*R*_	Min inhibitory concentration (MIC)	32
*k*_*R*_	Hill coefficient	4
