# Supplementary material for: Optimising Antibiotic Usage to Treat Bacterial Infections
Source: Sci Rep. 2016 Nov 28;6:37853. doi: 10.1038/srep37853 (PMC5124968; doi:10.1038/srep37853)
Supplement: Supplementary Information [file srep37853-s1.pdf]

Optimising Antibiotic Usage to Treat Bacterial Infections

Iona K. Paterson, Andy Hoyle, Gabriela Ochoa, Craig Baker-Austin, Nick G. H. Taylor

Supplementary Table S1 - Results from 1% initial resistant population

Table S1: Comparison of traditional dosage vectors (runs A, B, C and D), dosage vectors produced by the GA with deterministic modelling (runs E, F, G and K) and dosage vectors produced by the GA with stochastic modelling (runs H, I and J) for an infection with a resistant population of 1% of the total bacterial population.

Run	Dosage Vector	Total Antibiotic	Success Rate (%) [95% CI]
A	(21, 21, 21, 21, 21, 21, 21, 21, 21, 21)	210 $\mu g/ml$	99.5 [99.3, 99.7]
B	(21, 21, 21, 21, 21, 21, 21, 21, 21, 0)	189 $\mu g/ml$	98.4 [98.0, 98.7]
C	(21, 21, 21, 21, 21, 21, 21, 21, 0, 0)	168 $\mu g/ml$	96.7 [96.2, 97.2]
D	(21, 21, 21, 21, 21, 21, 21, 0, 0, 0)	168 $\mu g/ml$	85.4 [84.4, 86.4]
E	(60, 22, 4, 0, 0, 0, 0, 0, 0, 0)	86 $\mu g/ml$	86.9 [85.9, 87.8]
F	(50, 23, 13, 0, 0, 0, 0, 0, 0, 0)	86 $\mu g/ml$	87.2 [86.2, 88.1]
G	(40, 30, 20, 10, 0, 0, 0, 0, 0, 0)	100 $\mu g/ml$	91.8 [91.0, 92.5]
H	(60, 21, 10, 10, 2, 0, 0, 0, 0, 0)	103 $\mu g/ml$	96.2 [95.6, 96.7]
I	(50, 27, 19, 4, 0, 0, 0, 0, 0, 0)	100 $\mu g/ml$	96.4 [95.8, 96.9]
J	(40, 32, 18, 19, 0, 0, 0, 0, 0, 0)	109 $\mu g/ml$	96.2 [95.6, 96.7]
K	(60, 22, 22, 21, 15, 14, 10, 4, 0, 0)	168 $\mu g/ml$	99.9 [99.8, 100.0]

Supplementary Equations - Analytical Analysis of Antibiotic Free System

Using stability analysis the steady states of the system, in the absence of antibiotic, can be determined. At equilibrium, $dS/dt = dR/dt = 0$, there are four equilibrium points:

1) Extinction: $(S, R) = (0, 0)$

2) Susceptible Only: $(S, R) = (K(1 - \frac{\theta}{r}), 0)$

3) Resistant Only: $(S, R) = (0, K(1 - \frac{\theta}{r(1-a)})$

4) Co-existence: $(S, R) = (S^*, R^*)$ where

$$S^* = \frac{ar\theta + K\beta\,(ar - r + \theta)}{\beta\,(ar + K\beta)} \tag{1}$$

$$R^* = \frac{K\beta\,(r - \theta) - ar\theta}{\beta\,(ar + K\beta)} \tag{2}$$

Stability of the equilibrium points are found by calculating the Jacobian (Eq. 3) at each of the equilibria and calculating the corresponding eigenvalues.

$$J = \begin{pmatrix} r(1 - \frac{R+2S}{K}) - \beta R - \theta) & -\frac{rS}{K} - \beta S \\ -\frac{rR(1-a)}{K} + \beta R & r(1 - \frac{S+2R}{K})(1-a) + \beta S - \theta \end{pmatrix} \tag{3}$$

1) At the extinction equilibrium, $(0, 0)$, the Jacobian is reduced to Eq. 4.

$$J = \begin{pmatrix} r - \theta & 0 \\ 0 & r(1-a) - \theta \end{pmatrix} \tag{4}$$

The Jacobian matrix (Eq. 4) is a diagonal matrix and therefore the eigenvalues can be found on the diagonal. The extinction equilibrium is stable when Eq. 5 and 6 are satisfied.

$$r < \theta \tag{5}$$

$$r(1-a) < \theta \tag{6}$$

When the natural death rate is higher than the replication rate, for both the susceptible and resistant strains, the system will tend to extinction.

2) When evaluated at the resistant free equilibrium $(K(1 - \frac{\theta}{r}), 0)$, the Jacobian is reduced to Eq. 7.

$$J = \begin{pmatrix} \theta - r & \theta - \beta K - r - \frac{\beta K \theta}{r} \\ 0 & \beta K\left(1 - \frac{\theta}{r}\right) - \theta a \end{pmatrix} \tag{7}$$

Eq. 7 is upper triangular and therefore the eigenvalues can be found on the diagonal. For the resistant free equilibrium to be stable it must satisfy Eq. 8 and 9.

$$\theta < r \tag{8}$$

$$\beta K\left(1-\frac{\theta}{r}\right) < \theta a \tag{9}$$

The replication rate must be greater than the death rate otherwise the susceptible population would die out, therefore Eq. 8 must hold true. A lower transmission rate or a higher cost benefits the susceptible population.

3) Evaluating the stability at the susceptible free equilibrium $(0, K(1-\frac{\theta}{r(1-a)})$, the Jacobian is reduced to Eq. 10.

$$J = \begin{pmatrix} \frac{\theta}{(1-a)} - \beta K\left(1-\frac{\theta}{r(1-a)}\right) - \theta & 0 \\ \theta - r(1-a) + \beta K\left(1-\frac{\theta}{r(1-a)}\right) & \theta - r(1-a) \end{pmatrix} \tag{10}$$

Eq. 10 is lower triangular and the eigenvalues can be found on the diagonal. Therefore for the susceptible free equilibrium to be stable it must satisfy Eq. 11 and 12

$$\frac{\theta}{(1-a)} - \beta K\left(1-\frac{\theta}{r(1-a)}\right) - \theta < 0 \tag{11}$$

$$\theta < r(1-a) \tag{12}$$

The net replication rate must be greater than the death rate otherwise the resistant population would die out, therefore Eq. 11 must hold true. A higher transmission rate or a lower cost benefits the resistant population making it possible for the resistance bacteria to invade and out-compete an entirely susceptible population.

4) Analysis of the stability of the co-existence equilibrium is not possible due to the eigenvalues being analytically intractable. If it is hypothesised that stable co-existence is possible then from the previous equilibrium points it can be concluded that co-existence will occur, assuming a positive net growth rate for both bacteria, if:

$$\beta K\left(1-\frac{\theta}{r}\right) - \theta a > 0$$

and

$$\frac{\theta}{(1-a)} - \beta K\left(1-\frac{\theta}{r(1-a)}\right) - \theta > 0$$

Using the analytical analysis parameter values were chosen such that they satisfy Eq. 8 and 9. Therefore the resistant strain would not out-compete the susceptible strain in the absence of antibiotics.

Supplementary Figure S1 - Results from varied parameter values on success rate of regimens T3, S2 and S4

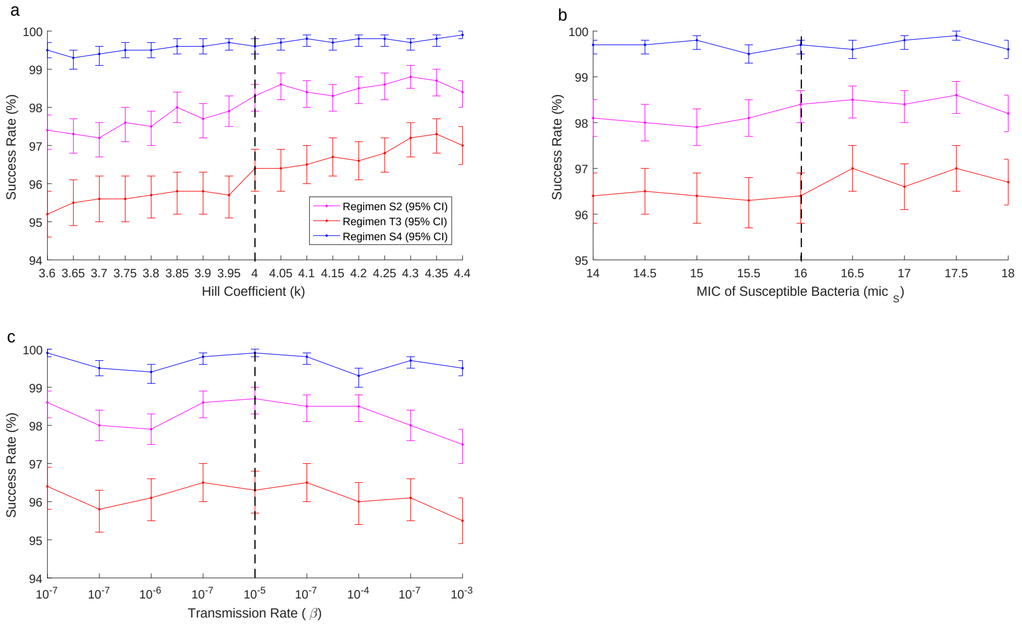

Figure S1: Success rates for regimens *S2* (pink), *T3* (red) and *S4* (blue) at varying values for parameters (a) k, (b) mic_S and (c) β. Black dashed line shows original parameter values. (a) Increasing k results in an increase in success rate for all 3 regimens. The difference in success rate between the 3 regimens remains consistent. (b) Altering the MIC of the susceptible bacteria has little effect on the success rate of the 3 treatment regimens. (c) Increasing the transmission rate of the resistant bacteria begins to decrease the success rate. The difference in success rate between the 3 regimens remains consistent.

Supplementary Table S2 - Results from varied weights within the objective function

Table S2: Comparison of dosage vectors produced by the GA with deterministic modelling, for varying values of w_1 and w_2.

w_1	w_2	Dosage Vector	Total Antibiotic	Success Rate (%) [95% CI]
0.5	0.5	$(60, 22, 23, 13, 0, 0, 0, 0, 0, 0)$	118 $\mu g/ml$	91.9 [91.1, 92.6]
		$(60, 22, 22, 14, 11, 0, 0, 0, 0, 0)$	129 $\mu g/ml$	95.0 [94.4, 95.6]
0.99	0.01	$(60, 22, 21, 15, 0, 0, 0, 0, 0, 0)$	118 $\mu g/ml$	92.3 [91.5, 93.0]
		$(60, 22, 18, 17, 11, 0, 0, 0, 0, 0)$	128 $\mu g/ml$	93.9 [93.2, 94.6]
